# *In vitro* and *in vivo* antitumour effects of coconut water vinegar on 4T1 breast cancer cells

**DOI:** 10.29219/fnr.v63.1616

**Published:** 2019-01-10

**Authors:** Nurul Elyani Mohamad, Swee Keong Yeap, Nadiah Abu, Kian Lam Lim, Nur Rizi Zamberi, Noraini Nordin, Shaiful Adzni Sharifuddin, Kamariah Long, Noorjahan Banu Alitheen

**Affiliations:** 1 Department of Cell and Molecular Biology, Faculty of Biotechnology and Biomolecular Science, Universiti Putra Malaysia, Serdang, Selangor, Malaysia; 2 China-ASEAN College of Marine Sciences, Xiamen University Malaysia, Jalan Sunsuria, Bandar Sunsuria, Sepang, Selangor, Malaysia; 3 Institute of Bioscience, Universiti Putra Malaysia, Serdang, Selangor, Malaysia; 4 UKM Medical Centre, UKM Medical Molecular Biology Institute (UMBI), Cheras, Wilayah Persekutuan, Malaysia; 5 Faculty of Medicine and Health Sciences, Universiti Tunku Abdul Rahman, Sungai Long Campus, Jalan Sungai Long, Bandar Sungai Long, Cheras, Kajang, Selangor, Malaysia; 6 Biotechnology Research Centre, Malaysian Agricultural Research and Development Institute (MARDI), Serdang, Selangor, Malaysia

**Keywords:** coconut water vinegar, breast cancer, metastasis

## Abstract

**Background:**

Coconut water and vinegars have been reported to possess potential anti-tumour and immunostimulatory effects. However, the anti-tumour, anti-inflammatory and immunostimulatory effects of coconut water vinegar have yet to be tested.

**Objective:**

This study investigated the in vitro and in vivo anti-tumour effects of coconut water vinegar on 4T1 breast cancer cells.

**Methods:**

The 4T1 cells were treated with freeze-dried coconut water vinegar and subjected to MTT cell viability, BrdU, annexin V/PI apoptosis, cell cycle and wound healing assays for the in vitro analysis. For the in vivo chemopreventive evaluation, mice challenged with 4T1 cells were treated with 0.08or 2.00 mL/kg body weight of fresh coconut water vinegar for 28 days. Tumour weight, apoptosis of tumour cells, metastasis and immunity of untreated mice and coconut water vinegar-treated 4T1 challenged mice were compared.

**Results:**

Freeze-dried coconut water vinegar reduced the cell viability, induced apoptosis and delayed the wound healing effect of 4T1 cells in vitro. In vivo, coconut water vinegar delayed 4T1 breast cancer progression in mice by inducing apoptosis and delaying the metastasis. Furthermore, coconut water vinegar also promoted immune cell cytotoxicity and production of anticancer cytokines. The results indicate that coconut water vinegar delays breast cancer progression by inducing apoptosis in breast cancer cells, suppressing metastasis and activating anti-tumour immunity.

**Conclusion:**

Coconut water vinegar is a potential health food ingredient with a chemopreventive effect.

## Popular scientific summary

- Coconut water has been associated with various health and medicinal benefits, including antioxidant, anti-inflammatory and immunostimulatory effects and these health benefits may be attributed to the presence of several bioactive compounds in its composition.
- The results of this study indicate that coconut water vinegar delays breast cancer progression by inducing apoptosis in breast cancer cells, suppressing metastasis and activating anti-tumour immunity.
- Coconut water vinegar is a potential health food ingredient with a chemopreventive effect.

## 

Cancer is a major public health issue and the second leading cause of death in developing countries. Despite intensive research and efforts, breast cancer incidence remains the most common cancer among women worldwide, and with the highest mortality rates. Advances in screening and breast cancer treatment are reported to reduce the incidence and mortality of breast cancer, and strategies, including maintaining a healthy lifestyle, can help reduce the breast cancer risk (1). Regarding a healthy lifestyle, evidence exists for the role of a healthy diet and physical activity in preventing and delaying cancer progression. For example, an Asian diet that is rich in plant-based food has been conveyed as a potential approach to reduce the risk and delay the progression of breast cancer (2). More specifically, an ethnic lifestyle and ethnic food, such as high consumption of soy-based products by Chinese women in South East Asia, may contribute to their higher 5-year overall survival compared to other ethnicities in Malaysia and Singapore (3). Besides soy-based products, the intake of many other food ingredients, such as vegetables, fruit and fibre, which have been recommended by the National Cancer Institute, also helps reduce the cancer risk (4).

Coconut (*Cocos nucifera* L.) is an important tropical fruit. Coconut water that is commonly consumed as a refreshing beverage in the tropical regions has been associated with various health and medicinal benefits, including antibacterial, antifungal, antiviral, anti-parasitic, anti-dermatophyte, antioxidant, hypoglycaemic and hepatoprotective benefits (5). These health benefits may be attributed to the presence of several bioactive compounds in its composition, including vitamins, amino acids, organic acids, enzymes (6) and phenolic acids (7). Coconut water has also been linked with anti-inflammatory (8) and immunostimulatory effects (9). In addition, peptides isolated from coconut water have been suggested as potential anticancer agents (9). Given that cancer has been identified as a disease of uncontrollable cell growth, associated with chronic inflammation and an immunosuppressive tumour microenvironment (10), coconut water, with its anti-inflammatory, immunostimulatory and cytotoxic activities (8, 9), may be beneficial in delaying cancer progression.

However, fresh fruit and vegetables have a limited shelf life. To overcome this limitation, fruit and vegetables can be fermented to prolong the shelf life or even enhance the availability of several bioactive components (11). Vinegar is a natural food additive, which is produced from fruits or vegetable rich in glucose, by a two-step process: alcohol fermentation and acetic acid fermentation. The common use of vinegar as a food seasoning and therapeutic agent is well established (12). Vinegar has been reported as an effective anti-obesity and anti-hyperglycaemic agent, mainly due to the presence of acetic acid and phenolic compounds (12, 13). Moreover, a previous study correlated the consumption of vinegar with prevention of oesophageal cancer (14). In other works, vinegar from unpolished rice demonstrated *in vitro* cytotoxic effects on squamous carcinoma (15) and *in vivo* anti-colon tumour effects (16). Also, sugar cane vinegar was reported to kill leukaemia cells via induction of apoptosis (17). Guo et al. (18) noted that vinegar prevented the formation of N-nitroso compounds, which are known carcinogens. These studies (15–18) helped justify the correlation of the use of vinegar with reduced cancer risk (14).

Vinegar can be produced from various sources of fruit and vegetables (12, 13). Although acetic acid is the main component in all types of vinegar, the health benefits of different types of vinegar may vary due to variations in the levels of antioxidants from both the source of carbohydrate and bacterial strains used in alcohol and acetous fermentation (19). Sugar-rich coconut water (6) is commonly used to produce vinegar. However, the bioactivities, particularly the antitumour effect on breast cancer, of this coconut water vinegar have not yet been tested. Thus, this study aimed to evaluate the *in vitro* and *in vivo* antitumour effects of coconut water vinegar on murine 4T1 breast cancer cells. In addition, the role of the anti-inflammatory and immunostimulatory influences of the coconut water vinegar that may indirectly contribute to the antitumour effects was also assessed.

## Materials and methods

### Preparation of coconut water vinegar

Coconut water vinegar was prepared according to a previous study (20). Pure and fresh coconut juice was bought from the local market in Malaysia (Pasar Borong, Selangor). The coconut juice was first fermented using *Saccharomyces cerevisiae 7013 INRA* to produce alcohol and then further fermented with *Acetobacter aceti vat Europeans* to give the final product, acetic acid. The sample was then left to mature at room temperature for 1 month and finally kept in a glass container at 4°C until use. For the *in vitro* study, coconut water vinegar was freeze-dried and stored frozen at −20°C. Before cell treatment, the freeze-dried coconut water vinegar was diluted using RPMI-1640 media, titrated to pH 7 and filtered through a 0.25 µm syringe filter.

### Cell culture

Mouse breast cancer cell line (4T1) was purchased from the American Type Cell Culture (ATCC) collection (ATCC, Rockville, MD, USA). The cells were preserved in RPMI containing 10% foetal bovine serum (FBS) and were grown at 37°C in a humidified incubator containing 5% CO_2_.

### 3-(4,5-Dimethyl-2-thiazolyl)-2,5-diphenyl-2H tetrazolium bromide (MTT) assay

Cytotoxicity of coconut water vinegar on murine 4T1 breast cancer cells was measured by the MTT assay. Firstly, the cells (0.8 × 10^5^ cells/well) were seeded in a 96-well plate overnight. Then, a twofold serial dilution of coconut juice vinegar was pipetted to the plate and incubated for 48 h. Afterwards, the MTT solution was added to all the wells and the plates were incubated for 3 h to allow the formation of formazan crystals. Then, the formazan crystals were solubilised by DMSO (Fisher Scientific, New Hampshire, USA) and the absorbance [optical density (OD)] was measured using an enzyme-linked immunosorbent assay (ELISA) plate reader (Bio-Tek Instruments, Vermont, USA) at 570 nm. The assay was done in triplicate, and the cytotoxicity effect of coconut juice vinegar was analysed using the following formula:

Cell viability (%) = (OD sample/OD control) × 100% (1)

### 5-Bromo-2´-deoxyuridine (BrdU) cell proliferation assay

The BrdU proliferation assay was measured using a BrdU cell proliferation kit (Merck, Germany). Firstly, the 4T1 cells (8 × 10^4^ cells/mL) were seeded in the plates and incubated overnight. The next day, the cells were treated with two different concentrations (IC_50_=0.27 mg/mL; IC_75_=0.35 mg/mL) of coconut juice vinegar, added with 20 μL of BrdU and incubated for another 72 h. Later, the cells were fixed and denatured using a fixing solution and stored at 4°C. The plates were washed and then incubated with 100 μL of the detector antibody per well for 1 h. Next, 100 μL of goat anti-mouse immunoglobulin G-horseradish peroxidase (HRP) conjugate was added for 30 min, followed by further incubation with 100 μL of the 3,3′,5,5′-tetramethylbenzidine substrate for 30 min. Finally, 100 μL of stop solution was added to the plates, and the absorbance was measured at 450 nm, using a μQuant plate reader (Bio-Tek Instruments, Vermont, USA).

### Cell cycle analysis

Cells were cultured at 2.3 × 10^5^ cells/well and treated with coconut juice vinegar at two different concentrations (IC_50_=0.27 and IC_75_=0.35 mg/mL) for 48 h. The cells were trypsinised and centrifuged at 2,000 rpm for 5 min. The pellet was collected and fixed with 70% ethanol for 1 week. Lastly, the fixed cells were stained with propidium iodide (PI) and analysed using fluorescence-activated cell sorting (FACS) flow cytometry (Becton Dickinson, New Jersey, USA).

### Annexin V analysis

The effect of coconut juice vinegar on apoptosis of the cells was measured using fluorescein isothiocyanate (FITC)-labelled annexin V antibody by flow cytometry, according to the manual protocol of the FITC annexin V apoptosis detection kit (BD Biosciences, California, USA). Briefly, the cells (2.3 × 10^5^ cells/well) were treated with coconut juice vinegar at 0.27 and 0.35 mg/mL, respectively, for 48 h. Then, the cells were double-stained with PI and FITC for 15 min in the dark and analysed using a FACSCalibur flow cytometer (Beckman Coulter, California, USA). The experiment was repeated in triplicate.

### Wound healing assay

The 4T1 cells were seeded in a 6-well plate overnight to reach full confluency. Then, a scratch was introduced in the middle of the well by using a sterile yellow tip, and the cells were incubated with IC_25_ (120 µg/mL) of freeze-dried coconut water vinegar for 24 h. A well with a scratch but without treatment was used as the control. After 24 h, the percentage of wound closure was calculated using the following formula:

Wound closure (%) = [(width of wound at 0 h – width of wound at 24 h)/width of wound at 0 h] × 100% (2)

### Animals

The female BALB/c mice (*n* = 32; age 4–6 weeks; average weight 20–22 g) were purchased from the Institute of Bioscience, Universiti Putra Malaysia. The mice were acclimatised and were given distilled water and standard pellet diet *ad libitum.* All procedures were performed according to the guidelines of the Animal Care and Use Committee, Universiti Putra Malaysia (UPM/FPV/PS/3.2.1.551/AUP-R168).

### In vivo antitumour effect of coconut water vinegar

The study was conducted according to Yeap et al. (21), with a slight modification. Dosages of the coconut water vinegar were derived from previous studies, where 0.08 mL/kg body weight (BW) was based on 1 Tbsp of vinegar in 250 mL of water, while 2 mL/kg BW has shown *in vivo* antioxidant and anti-inflammatory effects (20, 22). Initially, the acclimatised female mice (*n* = 32) were randomly divided into four groups with eight mice per group. The mice were pretreated with either distilled water or coconut juice vinegar for 6 weeks before being induced with 4T1 cells [1 × 10^5^ cells in 100 μL phosphate-buffered saline (PBS)] via subcutaneous injection on week 7, except for the normal control group (G1). The treatment continued for another 28 days before all mice were sacrificed (week 11).

G1 (*n* = 8): Non-induced mice were given 100 µL distilled water once per day throughout the study (normal control);

G2 (*n* = 8): Induced mice were given 100 µL distilled water once per day throughout the study (negative control);

G3 (*n* = 8): Induced mice were pre-treated with coconut juice vinegar (100 µL of 0.08 mL/kg BW) once per day;

G4 (*n* = 8): Induced mice were pretreated with coconut juice vinegar (100 µL of 2 mL/kg BW) once per day.

The tumours, spleen and blood were collected from the mice. The tumours were weighed and washed in PBS before they were divided into several parts and kept in liquid nitrogen until use.

### Terminal dUTP nick end labelling (TUNEL) assay

The terminal deoxynucleotidyl transferase (TdT)-mediated dUTP nick end labelling assay was performed using a DeadEnd colourimetric apoptosis detection kit (Promega, Wisconsin, USA), according to the manufacturer’s protocol. In brief, tumours were excised and embedded on a slide. Then, the slides were deparaffinised in xylene twice for 5 min, followed by rehydration in decreasing concentrations of ethanol (100, 95, 85, 70 and 50%). Next, the slides were washed in PBS. To detect apoptotic cells, the slides were fixed in 4% paraformaldehyde and permeabilised using proteinase K and then fixed again in 4% paraformaldehyde. The slides were then equilibrated using the equilibration buffer and labelled using TdT. Next, the slides were blocked in hydrogen peroxide before being incubated with streptavidin HRP. The slides were then developed using 3,3′-diaminobenzidine, mounted in glycerol and viewed under a bright-field inverted microscope (Nikon, Japan). The degree of DNA fragmentation was measured based on the presence of dark brown cells (apoptotic cell indicator) against a light brown background.

### Metastasis analyses using clonogenic assay

The metastasis of 4T1 cells was investigated using a clonogenic assay and bone marrow test, respectively. Briefly, the mice were killed. The liver, spleen, lungs and legs were removed from the body under a sterile condition, washed with PBS and prepared according to the assay, respectively.

The clonogenic assay was done according to a previous study (23). In brief, the organs (liver, spleen and kidney) were chopped into small pieces (< 1 cm^3^). Then, each organ was incubated in PBS and collagenase for 20–30 min at 37°C, with thorough mixing every 5 min. Next, the solution was passed through a 70-mm cell strainer (SPL, Korea) and spun at 2,000 rpm for 10 min. The pellet was then washed with PBS and re-suspended in 10 mL suspension medium (RPMI 1640, 10% FBS, 1% penicillin-streptomycin, 60 µM 6-thioguanine). Six serial dilutions were made in a 6-well plate, and the plate was incubated for 10 days. On day 10, the plate was fixed with methanol, stained with crystal violet, and then the number of colonies was counted.

### Quantitative reverse transcription polymerase chain reaction (qRT-PCR) analysis

Equal amounts of total RNA were extracted using an RNeasy mini kit (Qiagen, Germany) and were reverse-transcribed to cDNA using an iScript™ cDNA synthesis kit (Bio-Rad, California, USA). The cDNA was then subjected to qRT-PCR analysis, for the intracellular adhesion molecule-1 (iCAM), c-myc, inducible nitric oxide synthase (iNOS), nuclear factor kappa B (NF-κB), glyceraldehyde phosphate dehydrogenase (GAPDH), California, β-actin and hypoxanthine phosphoribosyltransferase (HRPT), using an iQ5 real-time PCR machine (Bio-Rad, California, USA). The differential expression results were normalised and analysed using the housekeeping genes (GAPDH, β-actin and HRPT), by the iQ5 Optical System software. The sequences of the primers used in the study are given in Table 1.

**Table 1:** Primer sequences

**Target genes**	
c-Myc	Forward:5’- TGATGTGGTGTCTGTGGAGAA-3’
	Reverse:5’- CGTAGTTGTGCTGGTGAGTG-3’
ICAM-1	Forward:5’- TGCTCAGGTATCCATCCATCC-3’
	Reverse:5’- ACGGTGCCACAGTTCTCAA-3’
iNOS	Forward:5’-GCACCGAGATTGGAGTTC-3’
	Reverse:5’-GAGCACAGCCACATTGAT-3’
NFκβ	Forward:5’-CATTCTGACCTTGCCTATCT-3’
	Reverse:5’-CTGCTGTTCTGTCCATTCT-3’

**Reference genes**	

GAPDH	Forward:5’-TTCCAGCCTTCCTTCTTG-3’
	Reverse:5’- GGAGCCAGAGCAGTAATC-3’
β-actin	Forward:5’-GAAGGTGGTGAAGCAGGCATC-3’
	Reverse:5’-GAAGGTGGAAGAGTGGGAGTT-3’
HPRT	Forward:5’-CGTGATTAGCGATGATGAAC-3’
	Reverse:5’- AATGTAATCCAGCAGGTCAG-3’

### Western blot analysis of matrix metalloproteinase 9 (MMP9) and vascular endothelial growth factor (VEGF) protein levels and quantification of nitric oxide (NO) by the Griess assay

The tumours were harvested, snap frozen and kept at −80°C until use. For the extraction of protein, each tumour was weighed and ground in a mortar and pestle using liquid nitrogen. Then, it was lysed in radioimmunoprecipitation assay buffer [150 mM sodium chloride, 1.0% NP-40 or Triton X-100, 0.5% sodium deoxycholate, 0.1% sodium dodecyl sulphate (SDS) and 50 mM Tris, pH 8.0] added with protease inhibitor cocktail (Pierce, Thermo Fisher Scientific, New Hampshire, USA). Protein was quantified using the Bradford assay (Bio-Rad, USA). The protein samples were then either kept frozen at −80°C or freshly used for western blot analysis and the Griess assay.

Equal amounts of protein were separated by sodium dodecyl sulphate-polyacrylamide gel electrophoresis (SDS-PAGE) and then transferred to a nitrocellulose membrane (Pall Corporation, Ann Arbor, MI, USA) that was subsequently blocked with 5% non-fat milk (Bio Basic, New York, USA) overnight. Next, the membrane was washed with Tris-buffered saline (10 mM Tris, 140 mM NaCl, pH 7.6) containing 0.1% Tween-20 (TBST) and further incubated in primary antibody at 4°C for 1 h, followed by washing with TBST, before incubation with secondary antibody for another hour. It was then rewashed and incubated with HRP substrate for 10 min before it was viewed using a Chemidoc imager (UVP, California, USA). B-actin (Abcam, USA) was used as a housekeeping control. The results obtained were analysed using Vision Work LS Analysis software (UVP, California, USA).

The NO level in each tumour was quantified using the Griess assay (Invitrogen, California, USA), according to the manufacturer’s protocol.

### Immunophenotyping by flow cytometry

Briefly, the harvested spleen was washed and passed through a 70-mm cell strainer (SPL, Korea) in PBS. The supernatant was spun at 2,000 rpm for 15 min. The pellet was lysed using NH_4_Cl buffer. Then, the splenocytes were stained with four different antibodies (CD3 and CD4, CD3 and CD8, CD3 and NK1.1, F4/80) and analysed by FACSCalibur flow cytometry (Becton Dickson, BD, New Jersey, USA).

### Cytokine assay

Blood was collected from each mouse using BD Microtainer^®^ tubes (Becton Dickinson, New Jersey, USA) and spun at 10,000 rpm for 15 min. The resultant serum was kept at −20°C until use. On the allocated day, the serum was diluted 10-fold using an assay diluent buffer and assayed by the ELISA cytokine assay; interleukin-2 (IL-2), IL-1 beta (IL-1β), IL-10 and interferon gamma (IFNγ). All assays were performed according to the supplied mouse cytokine kit protocols (R&D Systems, Minnesota, USA).

### Lactate dehydrogenase (LDH) cytotoxicity assay

The LDH procedure was performed as described elsewhere (21). YAC-1 cells are murine lymphoma cells that are sensitive to cytotoxic immune cells, including CD3^+^CD8^+^ T and NK cells. Co-culture of the splenocytes with YAC-1 cells has been commonly used to evaluate the cytotoxicity of CD8 T and NK cells (24). The cytotoxicity activity of coconut juice vinegar was measured using a Cytotox 96 nonradioactive cytotoxicity assay kit (Promega, Wisconsin, USA). In brief, the spleen was harvested from the untreated or coconut water vinegar-treated mice, and the splenocytes were isolated and incubated with YAC-1 cells for 24 h. The ratios of splenocytes (effector spontaneous) to YAC-1 cells (target spontaneous) used in this study were 2:1 and 5:1. The cytotoxicity of the splenocytes on YAC-1 cells was tested by the Cytotox 96 nonradioactive LDH assay (Promega, Wisconsin, USA) according to the manufacturer’s protocol.

### Statistical analyses

Quantitative data were expressed as mean ± standard deviation (SD) and were analysed using one-way analysis of variance (ANOVA).The group means were compared by Duncan’s test. Statistical Package for the Social Sciences (SPSS) version 16.0 was used for all data analysis. *P*-values of <0.05 were considered statistically significant.

## Results

### Coconut water vinegar reduced viability, induced proliferation and delayed wound healing of 4T1 cells

**Fig. 1:**
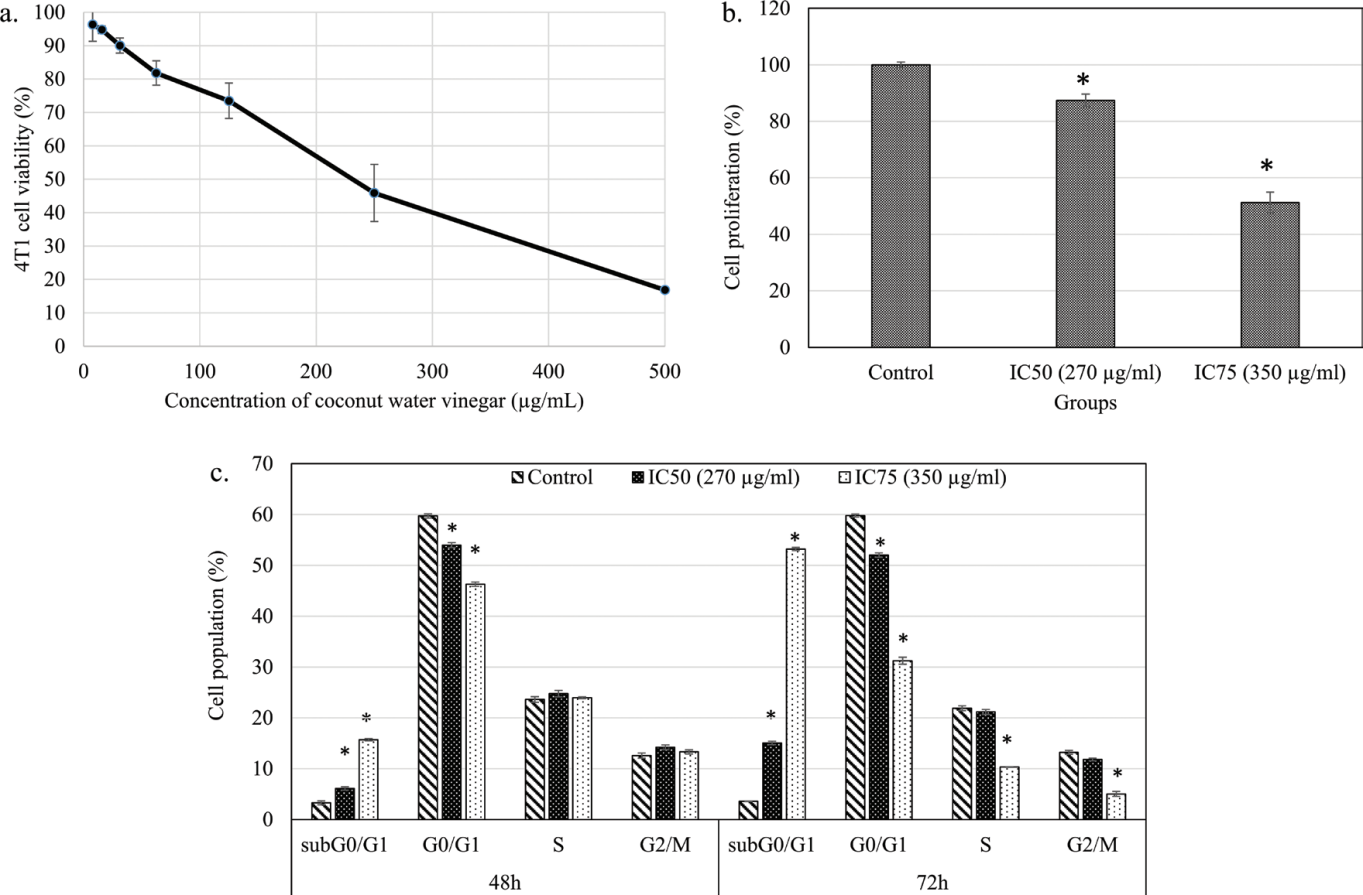
(a) The percentage of 4T1 cell viability after treatment with freeze-dried coconut vinegar for 48 h. (b) The percentage of 4T1 cell proliferation quantified by BrdU ELISA test after treatment with freeze-dried coconut water vinegar (IC_50_ and IC_75_) for 72 h. (c) Cell cycle progression of untreated and freeze-dried coconut water vinegar (IC_50_ and IC_75_)-treated 4T1 cells at 48 and 72 h. Data are expressed as mean ± SEM. *p < 0.05.

**Fig. 2:**
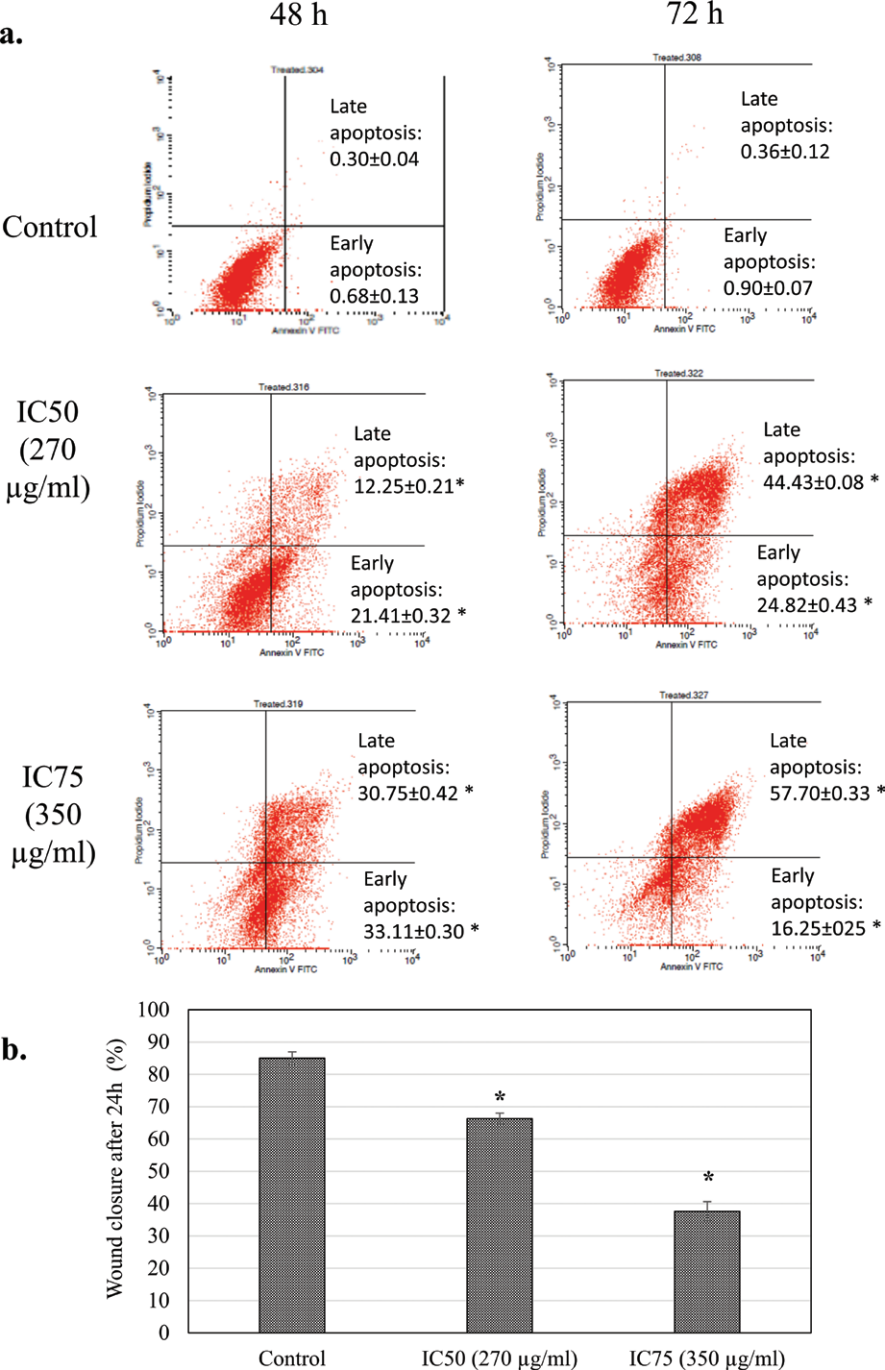
(a) Annexin V/PI apoptosis quantification of untreated and freeze-dried coconut water vinegar-treated 4T1 cells at 48 and 72 h. (b) The percentage of untreated and IC_25_ coconut water vinegar-treated 4T1 cell wound closure in the scratch test after incubation for 24 h. Data are expressed as mean ± SEM. *p < 0.05.

The MTT assay showed that freeze-dried coconut water vinegar lowered the viability of 4T1 cells in a dose-dependent manner after treatment at IC_50_ (270 µg/mL) and IC_75_ (350 µg/mL), respectively, at 48 h (Fig. 1a). The BrdU proliferation test showed that coconut water vinegar treatment at IC_50_ and IC_75_ reduced 13 and 49% cell proliferation, respectively, compared to the untreated 4T1 cells, at 72 h of incubation (Fig. 1b). Moreover, cell cycle profiling showed that at IC_50_, coconut water vinegar significantly (*p* < 0.05) decreased the G0/G1 phase-associated increase of the subG0/G1 phase at 48 and 72 h. At IC_75_, coconut water vinegar further reduced the population of cells in the S and G2/M phases, besides reducing those in the G1 phase and increasing cells in the subG0/G1 phase, at 72 h (Fig. 1c; Supplementary Fig. 1). Also, the annexin V/PI apoptosis assay showed that most of the 4T1 cells treated with coconut water vinegar at both IC_50_ and IC_75_ were under the early, rather than late, apoptosis stage at 48 h, and vice versa at 72 h (Fig. 2a).

After incubation for 24 h, the untreated 4T1 cells exhibited an approximate 80% wound closure in the scratch assay. Conversely, the coconut water vinegar treatment at IC_50_ and IC_75_ had around 66 and 37% wound closure in the scratch assay, respectively (Fig. 2b; Supplementary Fig. 2).

### Coconut water vinegar delayed tumour formation, induced apoptosis in tumour and inhibited metastasis of the tumour cells

**Fig. 3:**
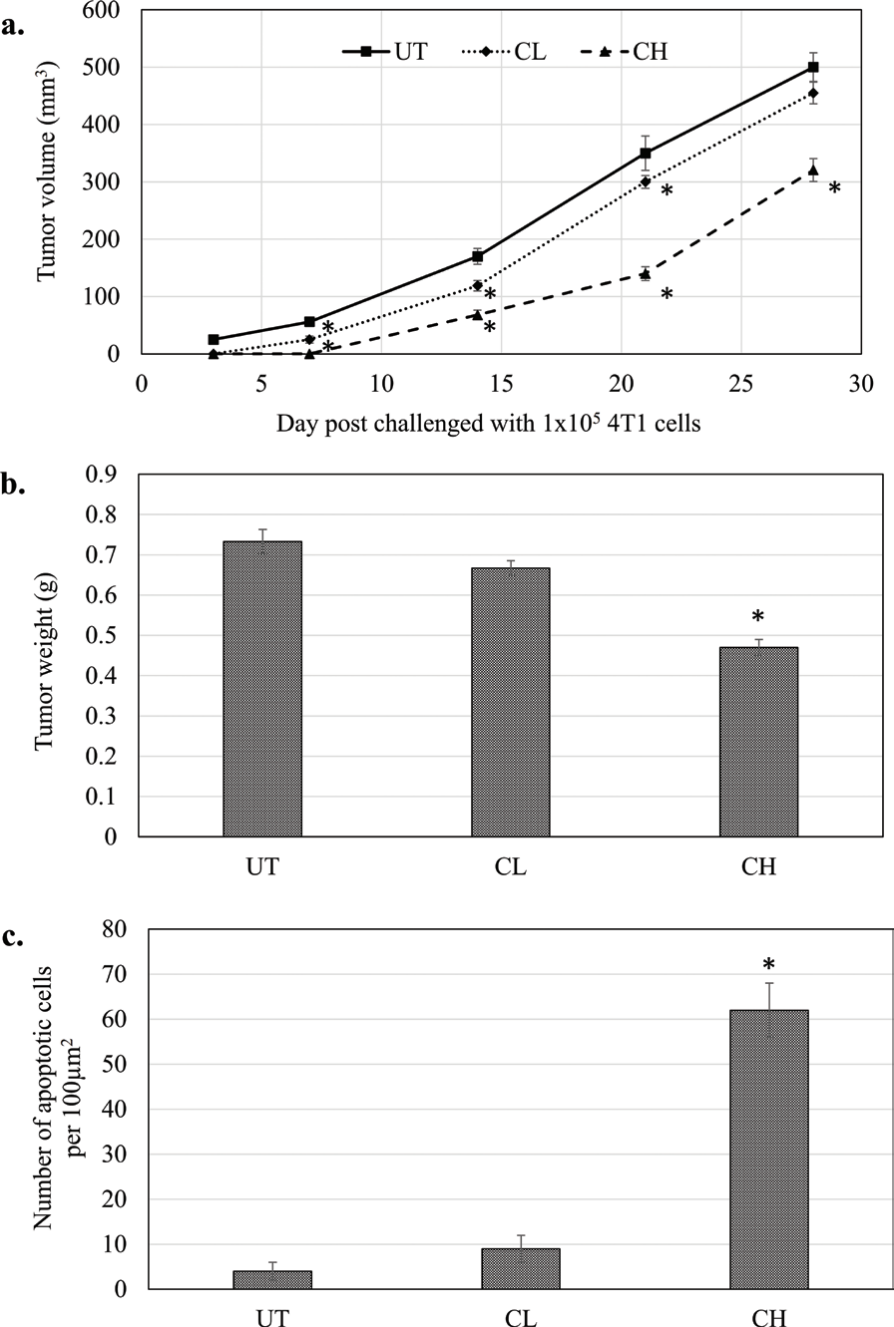
(a) Tumour volume (mm^3^) (from day 0 to 28 post-challenge) and (b) tumour weight at day 28 post-challenge of untreated, 0.08 (CL) and 2 (CH) mL/kg body weight coconut water vinegar-treated 4T1-challenged mice. (c) TUNEL assay quantification of the apoptotic event in the tumours of untreated, 0.08 (CL) and 2 (CH) mL/kg body weight coconut water vinegar-treated 4T1-challenged mice. Data are expressed as mean ± SEM. *p < 0.05.

*In vivo* treatment with coconut water vinegar, particularly at 2 mL/kg body weight (CH), exhibited delayed tumour formation, when mice post-challenged with 4T1 cells were compared to the untreated 4T1-challenged mice (Fig. 3a). At 28 days, the tumour size and weight of CH-treated mice were significantly (*p* < 0.05) lower than untreated mice post-challenged with 4T1 cells (Fig. 3a & b). In mice that received 0.08 mL/kg body weight (CL) of coconut water vinegar, tumour formation was also later relative to the untreated mice post-challenged with 4T1 cells. At 28 days, post-challenged CL mice were recorded with a lower but not statistically significant (*p* < 0.05) tumour volume and weight than untreated mice (Fig. 3a & b).

From Fig. 3c and Supplementary Fig. 3, it is clear that mice treated with CH displayed a high number of TUNEL-positive cells in the tumour immunohistochemistry (IHC). Likewise, the number of TUNEL-positive cells in the tumour of CL-treated mice was higher (though not significant) compared to the untreated mice.

**Fig. 4:**
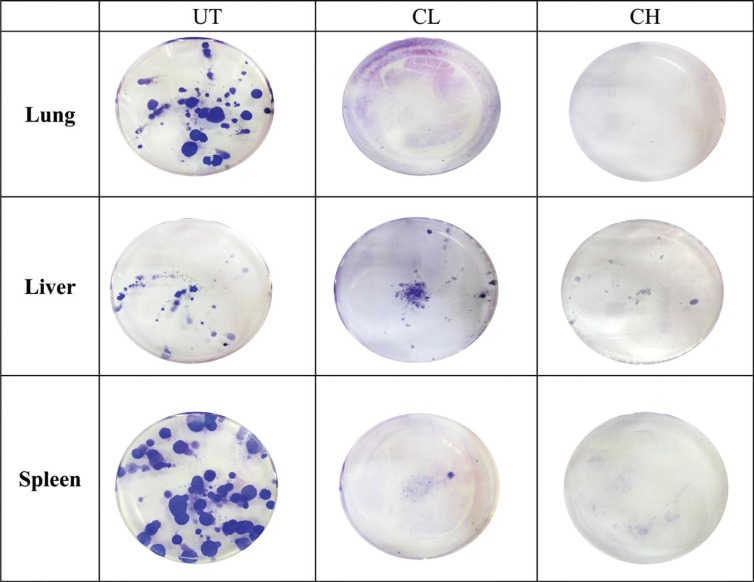
Representative image of the 4T1 colonies formed in the lungs, liver and spleen of untreated, 0.08 (CL) and 2 (CH) mL/kg body weight coconut water vinegar-treated 4T1-challenged mice.

In the CL-treated mice, metastasis of 4T1 cells was only observed in the liver but not in the lungs and spleen (single event). Regarding CH treatment, no metastasis was noticed in the liver, lungs and spleen (Fig. 4).

### Coconut water vinegar downregulated mRNA expression of iCAM, c-myc, iNOS and NF-κB, protein expression of VEGF and MMP9, and NO level in the tumour

**Fig. 5:**
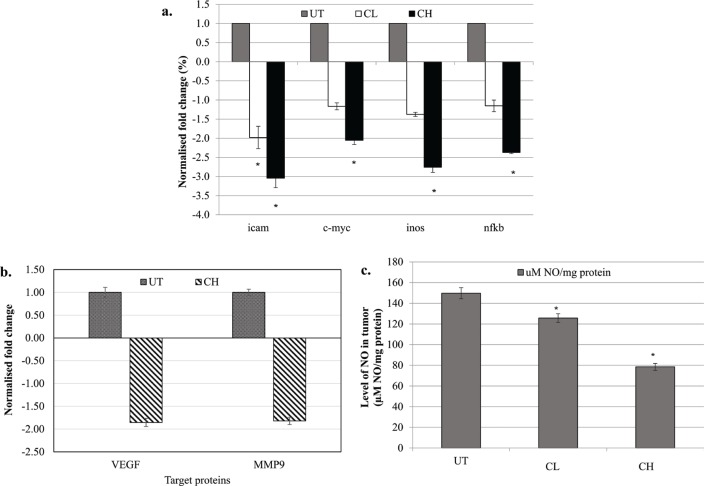
(a) Differential expression of iCAM, c-myc, iNOS and NF-κB genes in the tumour of untreated, 0.08 (CL) and 2 (CH) mL/kg body weight coconut water vinegar-treated 4T1-challenged mice quantified by qRT-PCR. Expression was normalised to GAPDH, HRPT and β-actin. (b) Differential expression of MMP9 and VEGF proteins in the tumour of untreated and 2 (CH) mL/kg body weight coconut water vinegar-treated 4T1-challenged mice quantified by western blot analysis. Expression was normalised to β-actin. (c) Nitric oxide (NO) level in the tumour of untreated, 0.08 (CL) and 2 (CH) mL/kg body weight coconut water vinegar-treated 4T1-challenged mice quantified by the Griess assay. Data are expressed as mean ± SEM. *p < 0.05.

Relative to the untreated mice, CL treatment only significantly (*p* < 0.05) downregulated the expression of the iCAM gene, while CH treatment downregulated the expression of all iCAM, c-myc, iNOS and NF-κB genes (Fig. 5a). In addition, differential protein expression of MMP9 and VEGF was measured using western blot analysis. CH treatment significantly (*p* < 0.05) reduced the protein expression of VEGF and MMP9 in the tumour of the mice compared to the untreated 4T1-challenged mice (Fig. 5b; Supplementary Fig. 4). The NO levels in the tumour of both CL- and CH-treated mice were significantly (*p* < 0.05) lower than that in untreated mice (Fig. 5c).

### Coconut water vinegar increased serum level of anti-cancer-related cytokines (IL-2 and IFNγ), spleen CD3^+^CD8^+^ and NK1.1 cells population and cytotoxicity of splenocytes

**Fig. 6:**
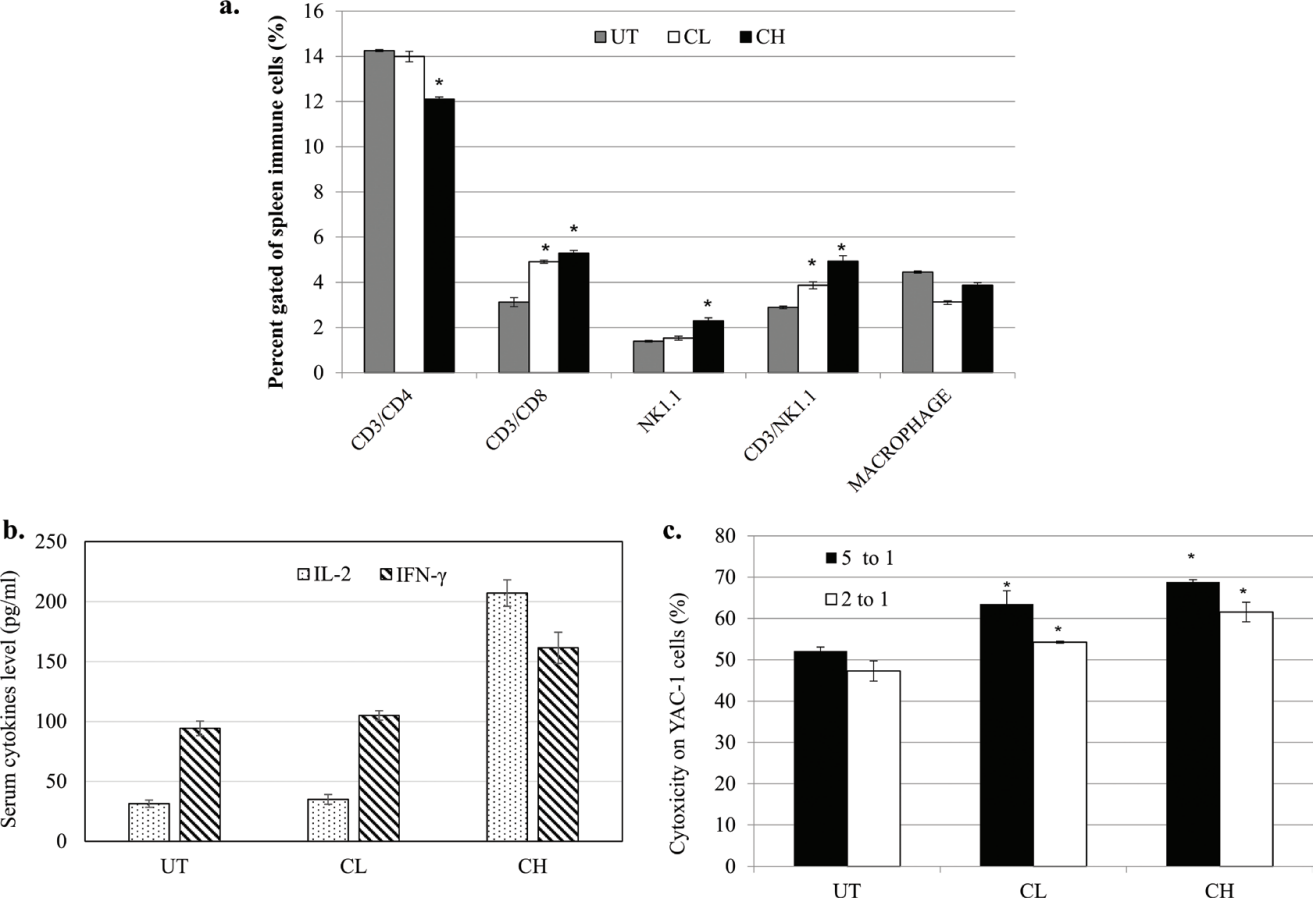
(a) Population of CD3^+^CD4^+^, CD3^+^CD8^+^, NK1.1 and macrophages in the spleen of untreated, 0.08 (CL) and 2 (CH) mL/kg body weight coconut water vinegar-treated 4T1-challenged mice quantified by flow cytometer immunophenotyping. (b) IL-2 and IFNγ levels in the serum of untreated, 0.08 (CL) and 2 (CH) mL/kg body weight coconut water vinegar-treated 4T1-challenged mice quantified by ELISA. (c) Co-cultivation of splenocytes from untreated, 0.08 (CL) and 2 (CH) mL/kg body weight coconut water vinegar-treated 4T1-challenged mice with YAC-1 cells at 5:1 and 2:1 ratios. Cytotoxicity was quantified by the Cytotox 96 non-radioactive LDH assay. Data are expressed as mean ± SEM. *p < 0.05.

The populations of cytolytic T lymphocyte (CD3^+^CD8^+^) and NK cells (NK1.1^+^) in the spleen of CL- and CH-treated mice were higher relative to the untreated 4T1-challenged mice. The helper T cell population (CD3^+^CD4^+^) and macrophages were slightly reduced in the spleen of CH-treated mice (Fig. 6a).

When compared with the untreated mice, the anti-cancer-related cytokines, IL-2 and IFNγ, occurred at a higher level in the serum of CH-treated mice, whereas the amounts were not significantly (*p* < 0.05) higher in the CL-treated mice (Fig. 6b).

Splenocytes harvested from untreated 4T1-challenged mice were recorded with about 50% cytotoxicity on murine YAC-1 lymphoma. Interestingly, both CL and CH treatments recorded higher cytotoxicity against YAC-1 cells at both 5:1 and 1:2 ratios of splenocytes-to-YAC-1 cells (Fig. 6c).

## Discussion

Breast cancer is still the most common type of cancer among women worldwide, and efforts in breast cancer prevention and treatment continue to receive considerable attention (1). A change in lifestyles, such as an increased intake of healthy food, especially fruit and vegetables, is believed to be an effective approach for cancer prevention (25). Chemoprevention by consumption of fruit and vegetables has been correlated with the presence of phytochemicals that possess antitumour effects (26). As mentioned above, coconut water has been associated with antitumour, immunostimulatory and anti-inflammatory effects (8–10). However, the antitumour effect of coconut water vinegar, which has been commonly consumed in tropical countries, has yet to be evaluated. Thus, this study was performed to assess its *in vitro* and *in vivo* antitumour effects on murine breast cancer 4T1 cells.

Freeze-dried coconut water vinegar reduced the viability, halted proliferation and induced apoptosis of 4T1 cells after 48 h of treatment *in vitro*. Previous reports have shown that various types of vinegar could kill human cancer cells via several modes (15, 17, 27). For example, sugar cane vinegar ‘kibizu’ induced apoptosis in human leukaemia cells (17) while unpolished rice vinegar ‘izumi’ induced death of human squamous cell carcinoma cells via necroptosis (15). Also, another type of unpolished rice vinegar ‘kurosu’ exhibited anti-proliferative and apoptosis induction effects in other human cancer cell lines, including colon Caco-2, lung A549, breast MCF-7, bladder 5,637 and prostate LNCaP cells (27). Comparing our results with the findings of these studies, it can be stated that different types of vinegar induce different modes of cell death in cancer cells, as previously proposed by Nishidai et al. (19). According to Nishidai et al., the variation of bioactivities displayed by the various types of vinegar may be attributed to the different levels of active metabolites that are present in the raw materials used for the fermentation. Active ingredients that present in the coconut water vinegar may contribute to the reduction of 4T1 cells viability via induction of apoptosis and delay the proliferation and closure of 4T1 cells, which need further studies.

Given that coconut water vinegar illustrated *in vitro* cytotoxicity in 4T1 cells, the *in vivo* antitumour effect was validated on the 4T1-challenged mice. Mice pretreated with 0.08 (CL) and 2 (CH) mL/kg body weight of coconut water vinegar, respectively, were observed with delayed tumour formation post-4T1 challenge in a dose-dependent manner. The results indicated that coconut water vinegar possessed an *in vivo* chemopreventive effect on breast cancer and delayed the progression of primary tumours in a dosage-dependent manner, which is similar to the anti-carcinogenetic impact of unpolished rice vinegar (16). The *in vitro* experiment revealed that induction of apoptosis in 4T1 cells was the main mode of cell death induced by coconut water vinegar. The IHC TUNEL assay result showed that the decreased tumour size recorded in CH-treated mice was because of the induction of apoptosis, which further supports the *in vitro* findings. In addition, the effective inhibition of primary tumour development by coconut water vinegar is possibly attributed to the suppression of c-myc gene expression, as seen in the qRT-PCR assay. C-myc is an oncogene that plays an important role in tumorigenesis (28). Coconut water vinegar treatment had little effect in controlling primary tumour development in CL mice, which might be explained by the non-significant downregulation of c-myc oncogene by the treatment.

Breast 4T1 cells are highly metastatic, triple-negative breast cancer cells and have been widely used as an aggressive murine breast cancer model that represents human stage 4 breast cancer (29, 30). Similar to an earlier report (29), metastasis of 4T1 cells was observed in the lungs, liver and spleen of the untreated mice. Some colonies of 4T1 metastatic cells were still noted in the liver of the CL-treated mice. Conversely, both CL and CH were effective in inhibiting metastasis in the lungs and spleen. The anti-metastatic effect is potentially due to the suppressed expression of iCAM, VEGF and MMP9 genes, as indicated by the qRT-PCR and western blot analyses. ICAM, VEGF and MMP9 are useful targets in treating aggressive breast cancer, as downregulation of these genes has been previously found to suppress breast cancer cell invasion and metastasis (31). Thus, downregulation of these targets collectively contributed to the effective inhibition of 4T1 metastasis by coconut water vinegar at both 0.08 and 2 mL/kg body weight.

Chronic inflammation is commonly found in the tumour microenvironment (10). NF-κB is the major inflammatory mediator, which also takes part in promoting tumour metastasis-related genes, including MMP-9, VEGF and iCAM. Overexpression of NF-κB is stated to stimulate iNOS, thereby producing an excessive NO amount (32), which subsequently promotes tumour growth via activation of c-myc, and induces metastasis by increasing VEGF production (31) in the presence of the p53 mutation. Considering that 4T1 are p53-deficient breast cancer cells (33), a high NO level, promoted by the NF-κB signalling pathway in the tumour of untreated 4T1-challenged mice, may contribute positively to the tumour progression and metastasis by inducing expression of c-myc, VEGF and MMP-9 genes. Previous research has shown that coconut water (34) and vinegar (35) possess an anti-inflammatory effect via inhibition of NF-κB gene expression. In the current study, CH treatment significantly (*p* < 0.05) suppressed the expression of NF-κB and iNOS genes, which contributed to the significantly (*p* < 0.05) lower NO level in the tumour of CH-treated mice compared to the untreated 4T1-challenged mice. This result suggests that the anti-inflammatory effect of coconut water vinegar at 2 mL/kg body weight helps suppress tumour progression through inhibition of the NF-κB signalling pathway and synthesis of NO.

An immunosuppressive effect of 4T1 cells has been reported in the literature (36). In that work, mice challenged with 4T1 cells were observed with dysfunction of cytotoxicity but without a significant reduction in the number of cytolytic T and NK cells. It subsequently supported the invasion of 4T1 cells into organs, including the spleen (36), as seen in the untreated 4T1-challenged mice in the present study. When treated with CH, the activation of immunity was observed, which was indicated by a higher population of cytolytic T cells and NK cells, higher levels of serum IL-2 and IFNγ cytokines and higher cytotoxicity against YAC-1 cells. IL-2 is the immunostimulatory cytokine that activates production of IFNγ, the proliferation of immune cells and the cytolytic activity of CD8 T and NK cells (37). Moreover, IFNγ is an anticancer cytokine that suppresses the progression and metastasis of cancer cells, including 4T1 cells (36). Increasing levels of IL-2 may directly contribute to stimulating proliferation, IFNγ and cytotoxicity of CD8 T and NK cells in the spleen of CH mice. The immunostimulatory effect of coconut vinegar treatment may also contribute to the suppression of tumour development and metastasis.

## Conclusions

In this study, coconut water vinegar demonstrated a dose-dependent antitumour effect in murine 4T1 breast cancer cells through induction of apoptosis in cancer cells, inhibition of cancer-associated inflammation and promotion of antitumour immunity. These actions delayed the progression of 4T1 breast cancer cells *in vivo*. Thus, we conclude that coconut water vinegar is a potential anticancer food supplement.

## Supplementary Material

*In vitro* and *in vivo* antitumour effects of coconut water vinegar on 4T1 breast cancer cells

*In vitro* and *in vivo* antitumour effects of coconut water vinegar on 4T1 breast cancer cells

*In vitro* and *in vivo* antitumour effects of coconut water vinegar on 4T1 breast cancer cells

*In vitro* and *in vivo* antitumour effects of coconut water vinegar on 4T1 breast cancer cells
