# Supplementary figures and images for: In vitro and in vivo antitumour effects of coconut water vinegar on 4T1 breast cancer cells
Source: Food Nutr Res. 2019 Jan 10;63:10.29219/fnr.v63.1616. doi: 10.29219/fnr.v63.1616 (PMC6387426; doi:10.29219/fnr.v63.1616)

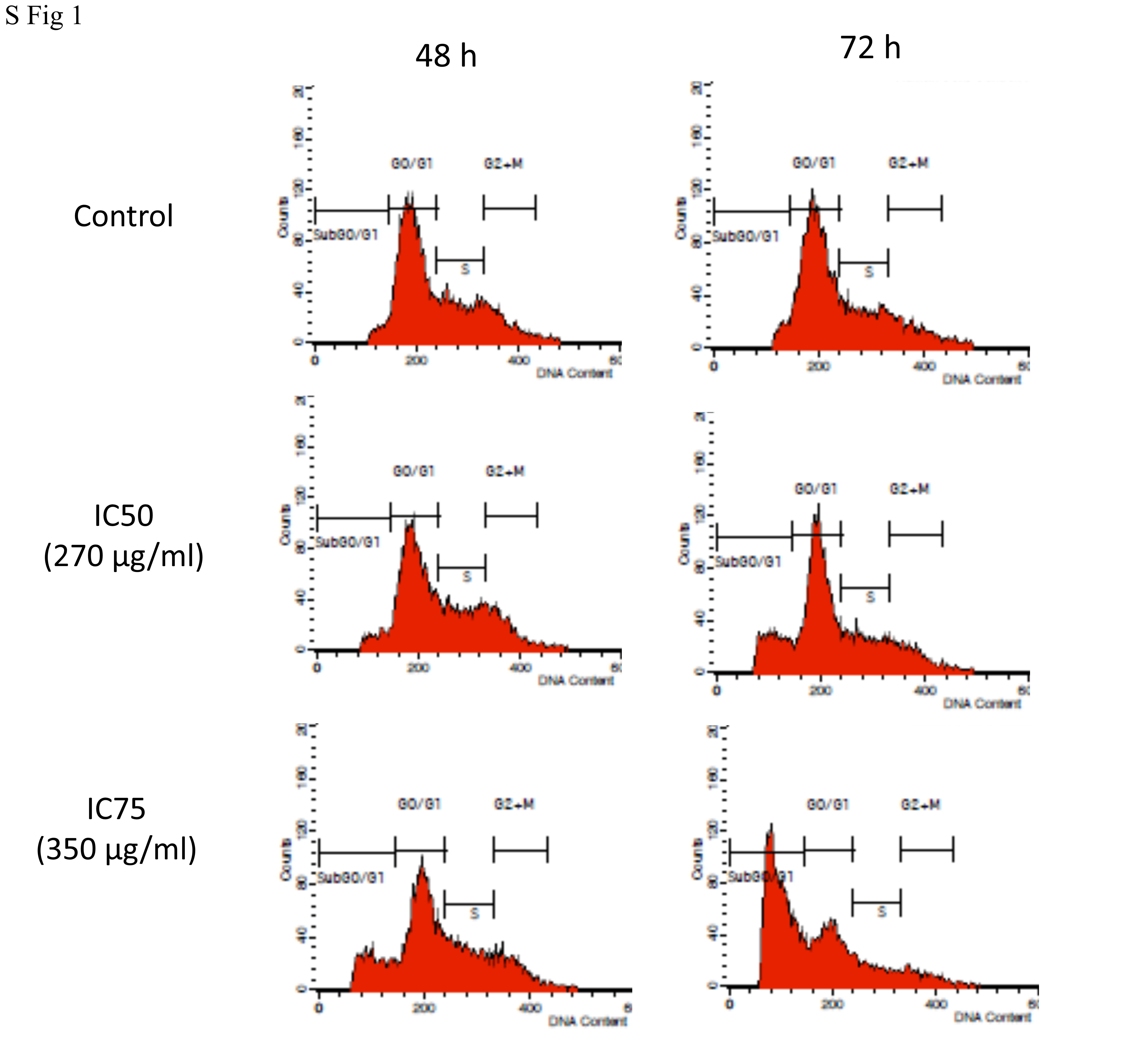

Supplement: In vitro and in vivo antitumour effects of coconut water vinegar on 4T1 breast cancer cells [file FNR-63-1616-s001.tif]

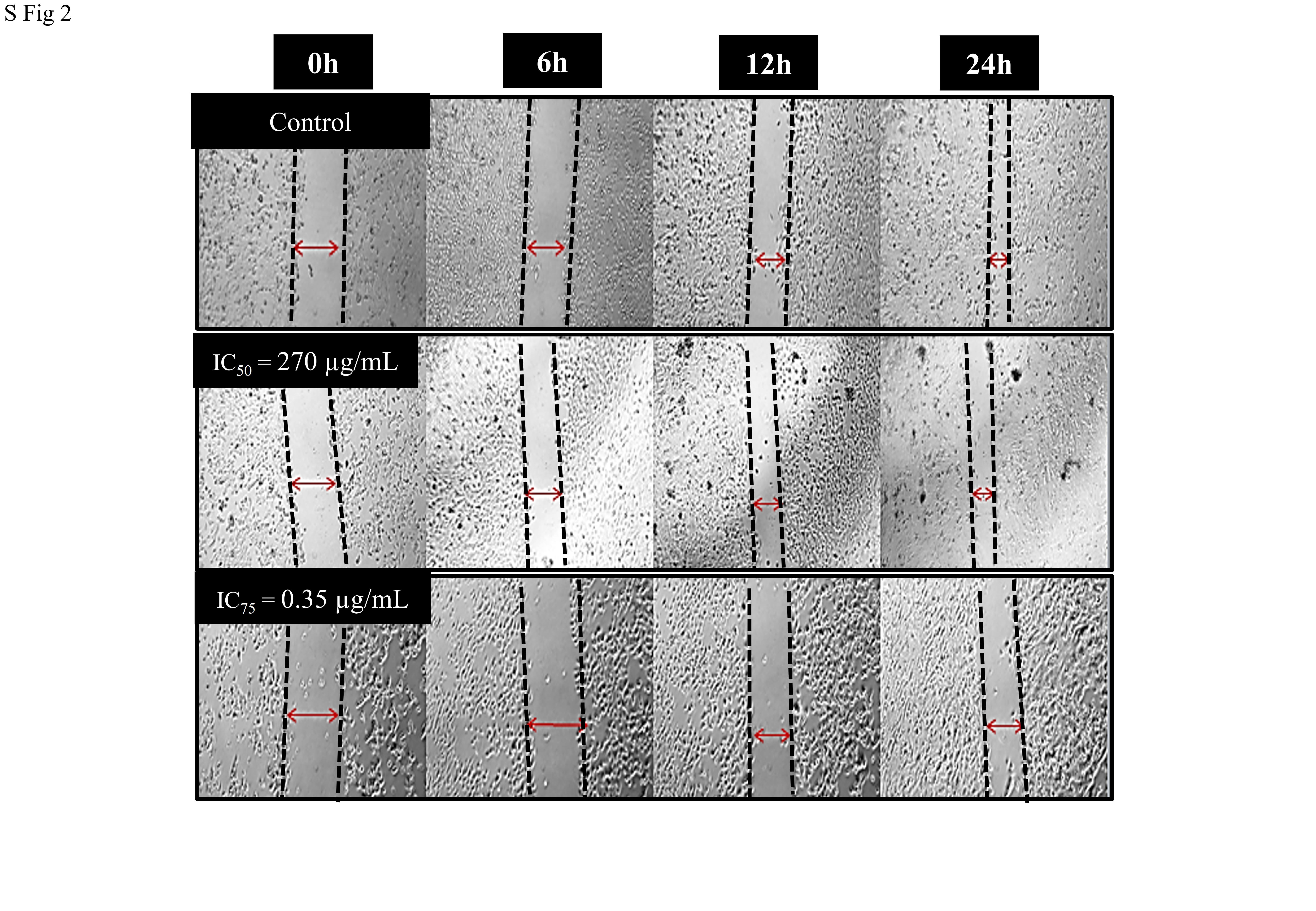

Supplement: In vitro and in vivo antitumour effects of coconut water vinegar on 4T1 breast cancer cells [file FNR-63-1616-s002.tif]

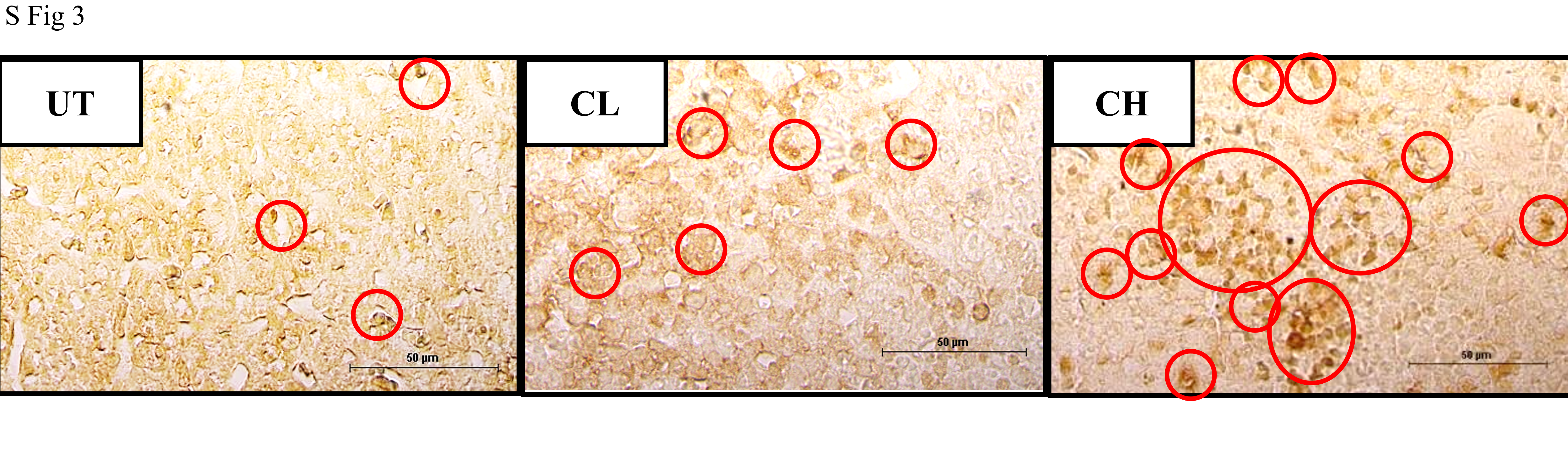

Supplement: In vitro and in vivo antitumour effects of coconut water vinegar on 4T1 breast cancer cells [file FNR-63-1616-s003.tif]

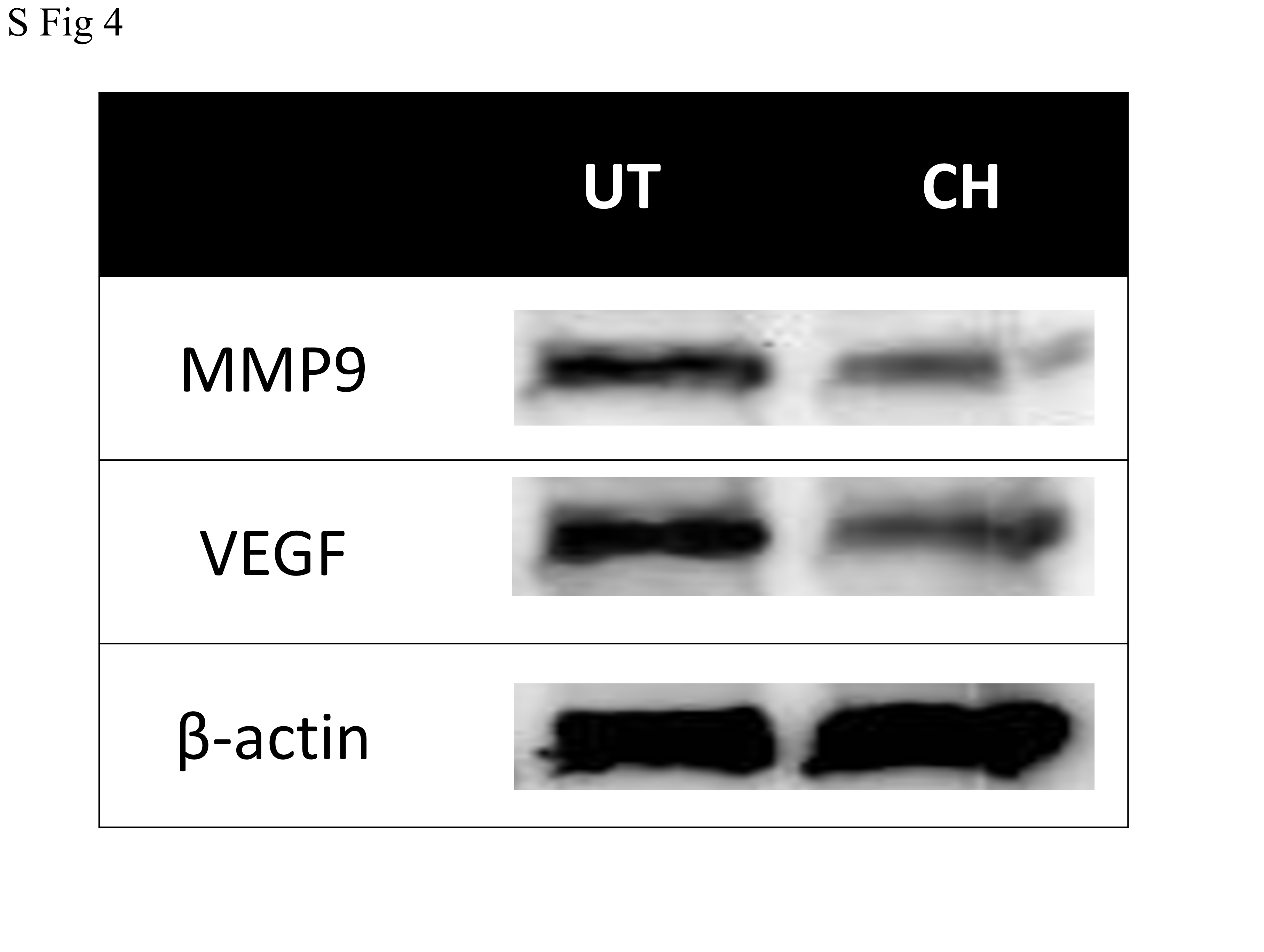

Supplement: In vitro and in vivo antitumour effects of coconut water vinegar on 4T1 breast cancer cells [file FNR-63-1616-s004.tif]
